# Advancements in the pathogenesis of hepatic osteodystrophy and the potential therapeutic of mesenchymal stromal cells

**DOI:** 10.1186/s13287-023-03605-z

**Published:** 2023-12-12

**Authors:** Senzhe Xia, Xueqian Qin, Jinglin Wang, Haozhen Ren

**Affiliations:** 1 Division of Hepatobiliary and Transplantation Surgery, Department of General Surgery, Nanjing Drum Tower Hospital Clinical College of Jiangsu University, Nanjing University, Nanjing, 210008 China; 2 Department of Oncological Surgery, Wenzhou Central Hospital, Wenzhou, 325000 China; 3 Division of Hepatobiliary and Transplantation Surgery, Department of General Surgery, Nanjing Drum Tower Hospital, The Affiliated Hospital of Medical School, Nanjing University, Nanjing, 210008 China

**Keywords:** Hepatic osteodystrophy, Chronic liver disease, Mesenchymal stromal cell, Scaffold material

## Abstract

Hepatic osteodystrophy (HOD) is a metabolically associated bone disease mainly manifested as osteoporosis with the characteristic of bone loss induced by chronic liver disease (CLD). Due to its high incidence in CLD patients and increased risk of fracture, the research on HOD has received considerable interest. The specific pathogenesis of HOD has not been fully revealed. While it is widely believed that disturbance of hormone level, abnormal secretion of cytokines and damage of intestinal barrier caused by CLD might jointly affect the bone metabolic balance of bone formation and bone absorption. At present, the treatment of HOD is mainly to alleviate the bone loss by drug treatment, but the efficacy and safety are not satisfactory. Mesenchymal stromal cells (MSCs) are cells with multidirectional differentiation potential, cell transplantation therapy based on MSCs is an emerging therapeutic approach. This review mainly summarized the pathogenesis and treatment of HOD, reviewed the research progress of MSCs therapy and the combination of MSCs and scaffolds in the application of osteoporotic bone defects, and discussed the potential and limitations of MSCs therapy, providing theoretical basis for subsequent studies.

## Background

Hepatic osteodystrophy (HOD) is a metabolically associated bone disease induced by chronic liver disease (CLD), which increases the risk of fracture and seriously affects the long-term prognosis of patients [1, 2]. CLD is liver necrosis and inflammatory disease caused by different pathogenesis and with a course of more than 6 months, which is the general terms of various chronic hepatic diseases, including nonalcoholic fatty liver disease, drug-induced liver injury, hepatic fibrosis, alcoholic liver disease, cirrhosis and hepatocellular carcinoma [3–5]. The incidence of osteodystrophy was positively correlated with the severity of chronic liver disease, and CLD patients have different degrees of bone changes in the early or late stages [6, 7]. Due to the pathological characteristics of osteopenia, the clinical symptoms of HOD are mainly osteoporosis and osteomalacia. Osteomalacia is the bone growth arrest caused by incomplete bone mineralization, and the incidence was relatively rare in clinic. Most HOD patients are characterized by osteoporosis, which is caused by the reduction of bone mass and the degradation of bone tissue microstructure, contributing to the change of bone fragility and increase the risk of fractures [8, 9]. At present, the internationally recognized bone mineral density (BMD) test method is dual-energy X-ray absorptiometry, and its T-value is the gold standard for diagnosis. The T-score indicates the difference between BMD and peak bone value of the same sex of the subject, and the T-score is 2.5 standard deviations below the normal reference value or more, and can be diagnosed as osteoporosis.

Worldwide, there is a large population of patients with viral cirrhosis [10], and the incidence of bone density changes in patients with viral cirrhosis varies greatly depending on the stage of disease progression. Bone density changes occur in 93.7% of patients with viral cirrhosis [11], which means that the majority of patients with viral hepatitis cirrhosis will develop bone changes sooner or later without intervention. In studies of bone changes in alcoholic fatty liver disease, excessive alcohol intake has been reported as an independent risk factor for the development of HOD. The prevalence of HOD is approximately 39.4% in patients with alcoholic liver disease [12]. Alcohol mainly promotes osteoclastic activity and enhances the differentiation of bone marrow-MSCs into adipocytes [13]. Primary biliary cirrhosis is a rare clinical cholestatic liver disease, which occurs mainly in female population [14]. However, it is noteworthy that HOD had a high incidence in primary biliary cirrhosis, reaching 37%, and indicated that the incidence of fractures reached 20.8% [15]. Liver transplantation is the most effective treatment for end-stage liver disease [16]. However, the use of immunosuppressants such as glucocorticoids after liver transplantation presented a further decline in BMD, and the bone damage associated with this drug tends to recover after the reduction of dose and the reconstruction of liver function [17, 18].

In order to reduce the adverse consequences of hepatic osteodystrophy, the current treatment of HOD mainly focuses on actively managing primary liver disease, adjusting unhealthy lifestyle and bone protective therapy. Regular supplementation of calcium and vitamin D3 is the cornerstone of bone protective drug therapy. In addition, antioxidant and anti-inflammatory therapy can improve the local bone microenvironment, alleviate bone and cartilage damage and promote regeneration. Moreover, it was pointed out that, on the basis of the above comprehensive treatment, the postmenopausal women with HOD could benefit from the combination of alendronate and risedronate to prevent or slow bone loss [19–21]. Raloxifen is a selective estrogen receptor modulator, which can prevent bone loss and reduce the risk of vertebral fracture [22]. It has been indicated that the use of raloxifene in primary biliary cirrhosis patients for 1 year significantly improved lumbar BMD without obvious adverse reactions [23]. Patients with osteoporosis often need treatment to alleviate the progress of the disease, including but not limited to drug treatment and biological treatment. Cell transplantation therapy can not only improve the neurological dysfunction of osteoporosis patients, but also have ability to reconstruct bone structure, improve BMD and tissue circulation, which was a novel method of therapy with increased attention and research [24–26]. Therefore, on the basis of previous studies, this review mainly summarized the pathogenesis of HOD, explored the function of MSCs in the treatment of osteoporosis and the strategy of MSCs engineering.

## The pathogenesis of hepatic osteodystrophy

The pathogenesis and pathological process of hepatic osteodystrophy are intricate and multifactorial, and have not yet been fully explored. Normal bone metabolism is a dynamic process of continuous remodeling of bone tissue, mainly including bone resorption and formation. In the bone metabolism, a certain amount of bone tissue is reabsorbed, and there will be a considerable amount of bone tissue synthesis, maintaining a dynamic balance under physiological conditions [27]. In the pathological situation of increased bone resorption or decreased bone formation, the bone metabolic balance is broken, and the bone mineral density gradually decreases to osteoporosis, and even leads to the occurrence of fractures [28, 29]. CLD is a systemic chronic disease that can cause hormonal imbalances, inflammatory factor release and other factors to disrupt bone metabolism balance, ultimately leading to the occurrence of HOD. At present, it is believed that the main mechanism of HOD is impaired function of osteoblasts and the broken balance of bone reconstruction, resulting in the reduction of bone formation rate.

The fluctuation of hormone level has ability to affect the balance of bone metabolism. Several studies have illustrated that androgen receptors and estrogen receptors are expressed in epiphyseal chondrocytes and growth plate cartilage, which indicates that sex hormones can affect bone growth and closure either directly or through local steroid receptors after hormone aromatization [30, 31]. At present, it has been indicated that estrogen or androgen participate in the regulation of bone metabolism balance mainly by inhibiting osteoclasts and alleviating the rate of bone absorption [32]. Estrogen can inhibit bone resorption by activating FasL gene expression to accelerate osteoclast apoptosis [33]. Similarly, androgens could exert directly or indirectly function on osteoclast progenitors and osteoclasts to decrease the number of osteoclasts and inducing apoptosis [34]. While estrogen receptors or androgen receptors were detected to be expressed on osteoblasts, and the effect of sex hormones on osteoblasts is to reduce cell apoptosis and participate in a series of metabolic processes such as osteoblast proliferation and bone matrix protein formation, thereby maintaining bone metabolism and protecting bone structure [35–37].

In CLD patients, the level of estrogen in the peripheral blood is relatively elevated due to the decreased ability of the liver to inactivate estrogen and the increased ability of androgen to convert into estrogen. As a result of this metabolic disorder, relatively elevated estrogen level can inhibit the hypothalamic-pituitary gonadal axis through the negative feedback system, resulting in reduced secretion of gonadotropin and adrenocorticotropin, and eventually lead to hypogonadism and decreased androgen levels. Although the level of estrogen may be higher than the level of androgen on the premise of liver metabolic dysfunction, both androgen and estrogen are significantly lower than the normal range, which is insufficient to inhibit osteoclasts, resulting in reduced bone formation and bone loss, especially in postmenopausal women with CLD, bone loss will be accelerated, and osteoporosis is more serious.

In addition to the effect of steroid hormone levels, serum cytokine levels in CLD patients were found to exert a role in the process of HOD. It was indicated that RANK/RANKL/OPG signaling pathway has a significant position in regulating bone metabolism and formation. As a soluble secretory glycoprotein, OPG was confirmed to have ability to inhibits bone resorption, while Rank can combine with RANKL on the surface of osteoclasts and precursor cells to strongly stimulate osteoclast differentiation and maturation, and inhibit osteoclast apoptosis [38, 39]. The hepatocytes of CLD patients are continuously injured, and chronic inflammation stimulates immune cells to produce various cytokines, which can affect bone metabolic balance directly or indirectly. IL-6, IL-1β and TNF-a are the main representative bone resorption cytokines, which can not only directly regulate the number and function of osteoclasts, but also promote the expression of RANKL in osteoblasts to indirectly enhance osteoclast activity [40–42]. However, the expression of these cytokines was confirmed to be increased in different types of CLD, so the crosstalk of cytokines may be one of the factors that accelerate the development of HOD [43–45]. Insulin-like growth factor-1 (IGF-1) is an important factor involved in bone formation, which is produced by the liver under the stimulation of growth hormone [46]. With the progression of CLD, the liver's biosynthetic function is weakened, and the synthesis of IGF-1 is restrained, leading to the rate of bone formation is inhibited [47]. Recent studies have found that increased phosphatase PP2Acα expression in HOD processes can reduce the expression of heparin lecithin cholesterol acyl transferase to affect cholesterol production and transport, and ultimately bone loss. Targeting the liver-bone axis present in HOD appears to achieve promising therapeutic results [1]. Hence, cytokine disturbances and inflammatory cytokine storms are significant elements in the occurrence and development of HOD, and targeting the relevant pathways seems to be beneficial.

**Fig. 1:**
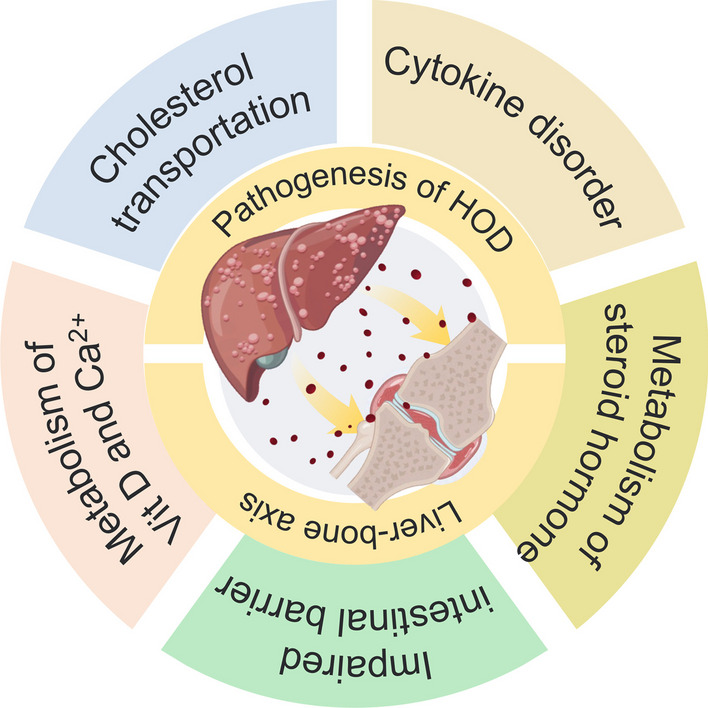
Pathogenesis of hepatic osteodystrophy. By Figdraw (www.figdraw.com)

Recently, research has found that intestinal barrier function and intestinal microorganisms also participate in the regulation of bone metabolism, and impaired intestinal barriers could lead to translocation of intestinal microbes and their metabolites, exacerbating systemic inflammatory responses. However, CLD is often accompanied by portal hypertension, which obstructs the return of intestinal blood flow and results in intestinal mucosal congestion, and the damaged intestinal barrier and increased permeability promote the translocation of intestinal microorganisms and their metabolites into the liver through the portal vein, and induce inflammation by activating Toll-like receptor signaling pathways on hepatic stellate cells and Kupfer cells, and produce a variety of inflammatory factors with bone absorption effect [48]. Moreover, it has confirmed that changes in the abundance and evenness of intestinal microflora in CLD patients can lead to multiple diseases related to metabolic abnormalities. Among them, secondary bone growth disorder may be related to the inhibition of anabolism caused by intestinal bacteria inhibiting IGF-1 signal pathway and increasing the expression of RANKL to promote bone absorption [49, 50]. The damage of the intestinal barrier can lead to the transfer of microorganisms or potential antigen to the epithelial submucosa, and disorder of immune cells in the intestinal and systemic immune responses cause the loss of bone mass [51]. In addition, intestinal microorganisms will also affect the absorption of vitamin D and vitamin K in the intestine, and indirectly disrupt bone metabolism [52]. Due to its complexity, the specific mechanisms of HOD pathogenesis have not been fully elucidated. Therefore, further exploration and summary of the potential mechanisms that may cause HOD development will facilitate timely clinical interventions to alleviate or terminate the progress of HOD (Fig. 1).

## The application of mesenchymal stromal cells in osteoporosis

In order to alleviate the progression of HOD and prevent the occurrence of complications, the current treatment is still actively treating primary liver disease and correcting unhealthy lifestyle. The existing drugs treatment of osteoporosis mainly include anti-absorbent and anabolic agents. Despite the symptoms of patients have been relieved after drug therapy, there are still concerns about the adverse reactions of anti-absorbent drugs and whether they can maintain long-term effectiveness. In recent years, numerous clinical researches have proved that MSCs have achieved considerable efficacy in the application of numerous diseases, and MSCs therapy has been recommended as a possible strategy for osteoporosis [53]. MSCs transplantation therapy can not only upgrade the neurological dysfunction of osteoporosis patients, but also reconstruct bone structure, improve bone mineral density and tissue circulation, which has attracted increasing attention and research [54]. At present, although there is little information on the preclinical or clinical application of MSCs in HOD, considering the effectiveness of MSCs in application of osteoporosis, cell therapy may have the potential to become a treatment direction for HOD.

MSCs transplantation is a promising therapeutic strategy, which has ability to enhance the differentiation of tissue-resident progenitor cells into osteoblasts and improve the balance of bone metabolism to achieve the purpose of treating osteoporosis [25]. Although there are few studies on MSCs in the treatment of HOD model, MSCs transplantation have been relatively mature method in the study of osteoporosis. The research and application of MSCs transplantation in improving osteoporosis have been relatively mature. At present, there are abundant sources of MSCs have been applied in the treatment and research of osteoporosis, mainly including bone marrow mesenchymal stromal cells (BM-MSCs), adipose mesenchymal stromal cells (AD-MSCs) and umbilical cord mesenchymal stromal cells (UC-MSCs). It has been confirmed that different sources of MSCs have significant therapeutic effects on osteoporosis [55]. Due to its excellent osteogenic differentiation ability and easy acquisition, BM-MSCs are the most commonly used seed cells in clinical or preclinical studies [25]. Ocarino Nde M et al. extracted BM-MSCs from healthy rats and transplanted them into a rat model of osteoporosis, and confirmed that transplantation of BM-MSCs could reverse osteoporosis by comparing the bone trabecular percentage [56]. Similar findings that transplantation of BM-MSCs can reduce bone mass loss and inhibit the progression of osteoporosis were also confirmed by Ichioka et al. [57]. Recently, AD-MSCs have been widely studied due to the advantages of AD-MSCs such as excellent differentiation ability, accessibility and abundance [58]. In addition, the proliferation and differentiation of AD-MSCs were less affected by age and multiple passage [59], and it was found that compared with BM-MSCs, AD-MSCs retained anti-inflammatory ability and could improve the bone microenvironment [60]. UC-MSCs mainly exist in the umbilical cord tissue of newborns and have obvious advantages in low immunogenicity and differentiation ability compared to BM-MSCs [61]. AN et al. confirmed that bone density was significantly improved after UC-MSCs transplantation, and the number and thickness of bone trabeculae were significantly increased [62]. Through the studies of transplantation of MSCs in the animal model of osteoporosis, it has revealed that the transplantation of MSCs can improve bone formation and maintain bone microstructure, while the bone quality and strength can also be improved [63, 64]. In the treatment of HOD induced by CLD, MSCs transplantation seems to achieve therapeutic effects in theory, but whether MSCs transplantation has an impact on primary liver disease still needs to be considered. Current studies have found that MSCs transplantation therapy has a certain therapeutic effect on acute or chronic liver diseases, reducing the occurrence of clinical complications without significant side effects [65–67]. Therefore, whether it is systemic or local transplantation, the efficacy and safety of MSCs transplantation therapy have theoretical basis. In theory, in addition to alleviating the symptoms of HOD, it can also have certain benefits in the treatment of CLD.

The therapeutic effect of MSCs transplantation has been confirmed, due to the limitations of low homing rate and low efficiency of osteogenic differentiation, various strategies have been used to modify MSCs to enhance its efficacy [68]. The ability of circulating MSCs or exogenous MSCs to locate and enter the corresponding injury site is known as the homing of MSCs, and the homing ability has a great impact on MSCs therapeutic effect [69]. Regardless of systemic or local administration, MSCs need to be homing to the lesion site to effectively exert their effect. However, the spontaneous homing efficiency of MSCs is quite low, which is one of the difficulties in the treatment of osteoporosis by MSCs [70]. Specific chemokine receptors exist on the surface of MSCs, while chemokines exist on the surface of endothelial cells [71, 72], which can activate homologous chemokine receptors on MSCs, thus enhancing the stimulative effect of integrin, making MSCs firmly adhere to endothelial cells and completing the homing process of MSCs [73]. After completion of homing activities, MSCs can favorably participate in bone tissue repair and regeneration through enhancing local osteogenic differentiation [55]. In addition, the studies have indicated that MSCs have ability to inhibit the differentiation of osteoclast precursor cell into osteoclasts to achieve the therapeutic effect of MSCs through inducing the secretion of osteoprotective hormone and the expression of WNT-1 at the cell level [74, 75]. These characteristics of MSCs regulating bone balance lay a foundation for the treatment of osteoporosis by MSCs.

**Fig. 2:**
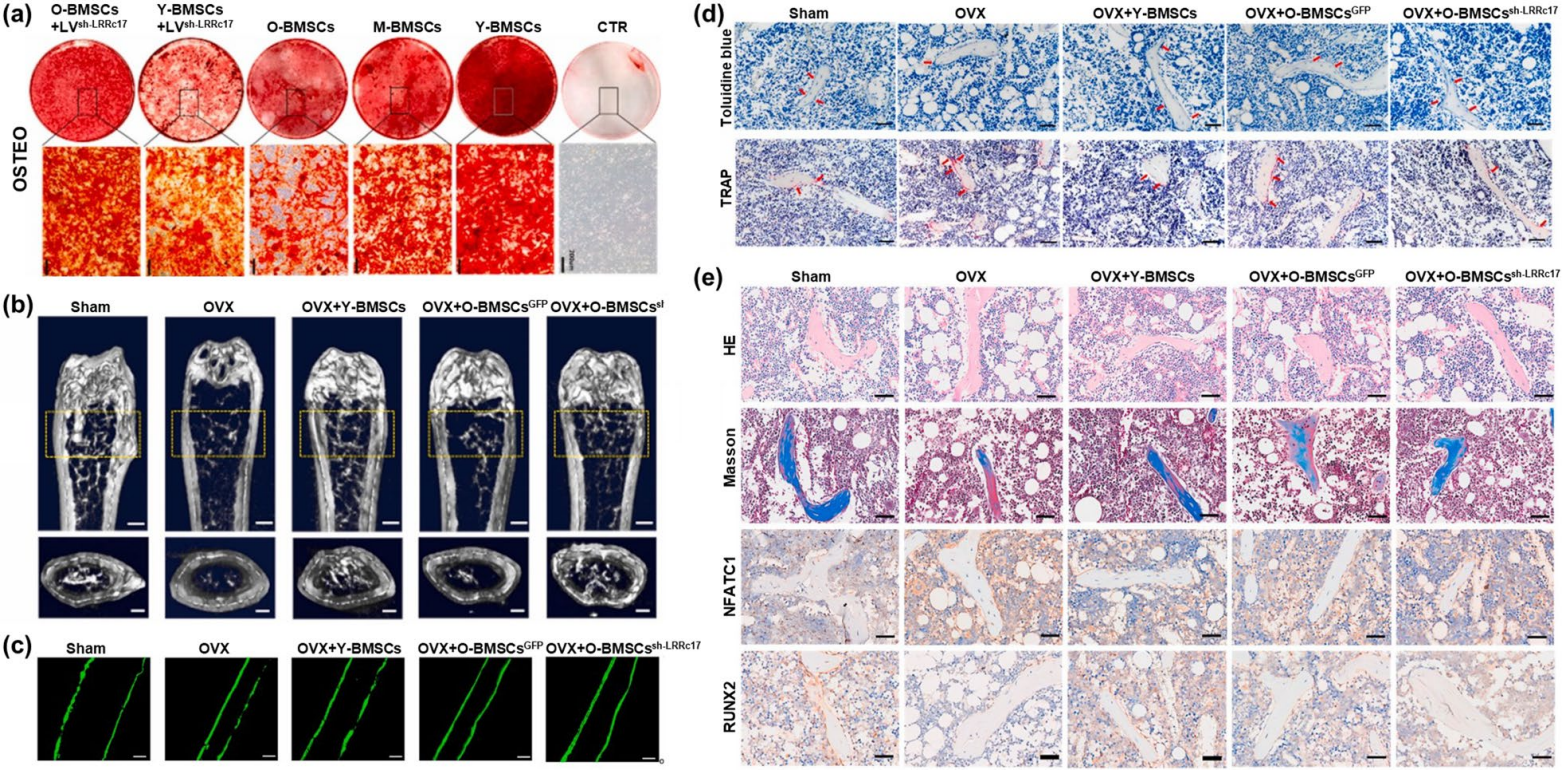
The knockout of LRRc17 can alleviate MSCs senescence and promote osteogenic differentiation. **a** The knockout of LRRc17 can alleviate the senescence of MSCs and enhance the osteogenic differentiation potential in vitro. **b**–**d** Transplantation of senescent MSCs with LRRc17 knocked out in osteoporosis mouse models can reverse the functional decline of MSCs caused by aging. **e** Transplantation of senescent MSCs with LRRc17 knocked out in osteoporosis mouse models can reduce bone defect area. Reproduced with permission from [79]

MSCs transplantation has demonstrated superior therapeutic effects such as immune regulation and regeneration, but the therapeutic mechanism of MSCs transplantation has not yet been determined. It is widely accepted that the regenerative effect of MSCs is to induce the regeneration and differentiation of tissue-resident progenitor cells mainly by secreting bioactive factors and nutrient mediators, including cytokines, growth factors and hormones, at the injured site or paracrine [76]. In addition, the active substances secreted by MSCs can be encased in extracellular vesicles to exert sustained function [68]. In order to improve the therapeutic effect of MSCs, gene modification, targeted modification, co-culture and co-transplantation are applied to program MSCs, among which gene modified MSCs transplantation is currently receiving widespread attention. Inducing overexpression of osteogenic differentiation related genes in MSCs is one of the common strategies for gene modification. Overexpression of homing-related genes has ability to the directed transport of MSCs and improved homing efficiency. It has been found that CXCR4 is involved in regulating the homing movement of MSCs, and CXCR4 upregulated MSCs can improve cell homing rate, thereby amplifying the therapeutic effect of MSCs, increasing vertebral bone density and biomechanical properties [77]. Based on the involvement of genes such as RUNX2, SP7, and BMP2 in regulating the differentiation of osteogenic, inducing overexpression of osteogenic related genes in MSCs can enhance the efficiency of directional differentiation of osteoblasts and improve therapeutic effectiveness compared to the control group [25, 78]. Moreover, in almost all clinical and preclinical transplants of MSCs, the short half-life of transplanted MSCs limits their efficacy. Hence, prolonging the life span of MSCs, delaying MSCs senescence and enhancing cell activity are also effective strategies. Liu et al. revealed that LRRc17 had ability to manipulated MSCs senescence and differentiation, and LRRc17 knockout can the apoptosis of MSCs, alleviate senescence and enhance the ability of MSCs to resist bone loss [79] (Fig. 2). Intriguingly, more studies have found that apoptosis of MSCs is essential for their therapeutic effects. It has been found that apoptosis occurs rapidly in the short term after transplantation of MSCs in vivo, and apoptotic bodies released during apoptosis are the main mediators of therapeutic effect [80, 81]. Furthermore, in order to explore the strategy of enhancing the activity of MSCs and prolonging the treatment time and effectiveness, assembling MSCs with biomaterials to generate engineered MSCs is a fairly interesting direction.

## The potential application of mesenchymal stromal cells combined with scaffold

**Fig. 3:**
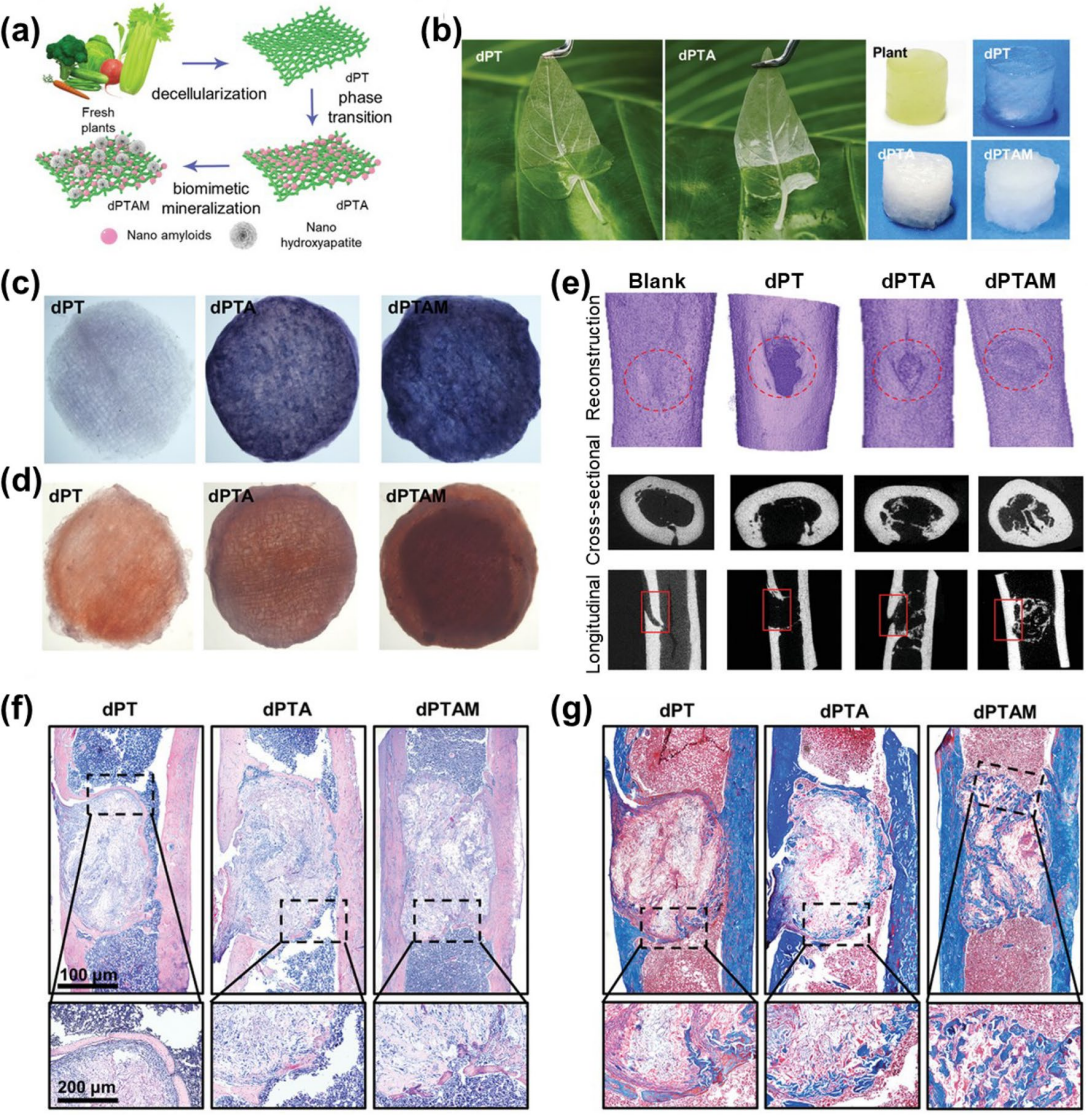
Natural plant tissue decellularized scaffolds can promote osteogenic differentiation in vivo and in vitro. **a**, **b** Diagram of the plant tissue scaffold preparation for extracting decellularized plant tissues (dPT), dPT coated with nano amyloids (dPTA) and dPTA mineralized to deposit nano-hydroxyapatite (dPTAM) from fresh plants; **c**, **d** The positive effect of nano amyloid protein/hydroxyapatite scaffold on osteogenic differentiation has been confirmed through in vitro experiments; (e–g) The safety of the scaffold and its ability to promote osteogenic differentiation in vivo were confirmed by the experiment of transplanting the scaffold to the bone defect site. Reproduced from Advanced healthcare materials, 11(12), Li Y, Fu Y, Zhang H, Wang X, Chen T, Wu Y, et al. Natural Plant Tissue with Bioinspired Nano Amyloid and Hydroxyapatite as Green Scaffolds for Bone Regeneration. e2102807, 2022, with permission from John Wiley and Sons [90]

The ability of MSCs to regulate bone balance and maintain bone mass has been demonstrated, and current research efforts are focused on engineering MSCs to impart their special performance and then apply them in the treatment of osteoporosis. MSCs are mainly used in the treatment of osteoporotic bone defect through cell transplantation, which mainly includes local implantation, systemic implantation and combined biological scaffold implantation [82, 83]. The cell activity of MSCs can be enhanced and the short half-life of MSCs in vivo transplantation can be overcome to a certain extent after combined with biological scaffolds. Hence, the selection of different scaffold materials has become the focus of research [68, 84]. Hydrogel system is regarded as a method of controlled release system, Tzouanas et al. confirmed that MSCs coated with hydrogel system have superior cell viability and prolong the action cycle [85]. Moreover, the culture environment in vitro has a significant impact on the differentiation and function of MSCs [86]. It has been found that the mechanical properties of the environment and the stiffness of the attached matrix have important effects on the proliferation and differentiation of MSCs [87]. The shear force and tension of extracellular fluid promoted the osteoblastic differentiation of MSCs in vitro, and the increase of hydrostatic pressure increased the expression of chondroblast genes and promoted chondrogenic differentiation [88]. The mechanical properties of scaffolds are the inducible factors for directional differentiation of MSCs, and the mechanical properties of various suitable materials are different. It has found that 3D decellularized scaffolds derived from natural plant tissue supported MSCs to differentiate into osteoblasts with high expression of ALP, Osteocalcin and RUNX-2, and that the performance of the scaffolds was improved by coating nano amyloids, or by further biomimetic mineralization deposition of nano-hydroxyapatite [89, 90] (Fig. 3). In addition, researchers also focused on the ability of scaffold to coat and release drugs. Epigallocatechin-3-gallate is a compound that can induce the differentiation of MSCs into osteoblasts. Wang et al. coated Epigallocatechin-3-gallate with chitosan/alginate scaffolds to form composite scaffolds, which have ability to release Epigallocatechin-3-gallate slowly and continuously, thus enhancing the bioavailability of Epigallocatechin-3-gallate to MSCs and the efficiency of MSCs differentiation into osteoblasts [91].

**Fig. 4:**
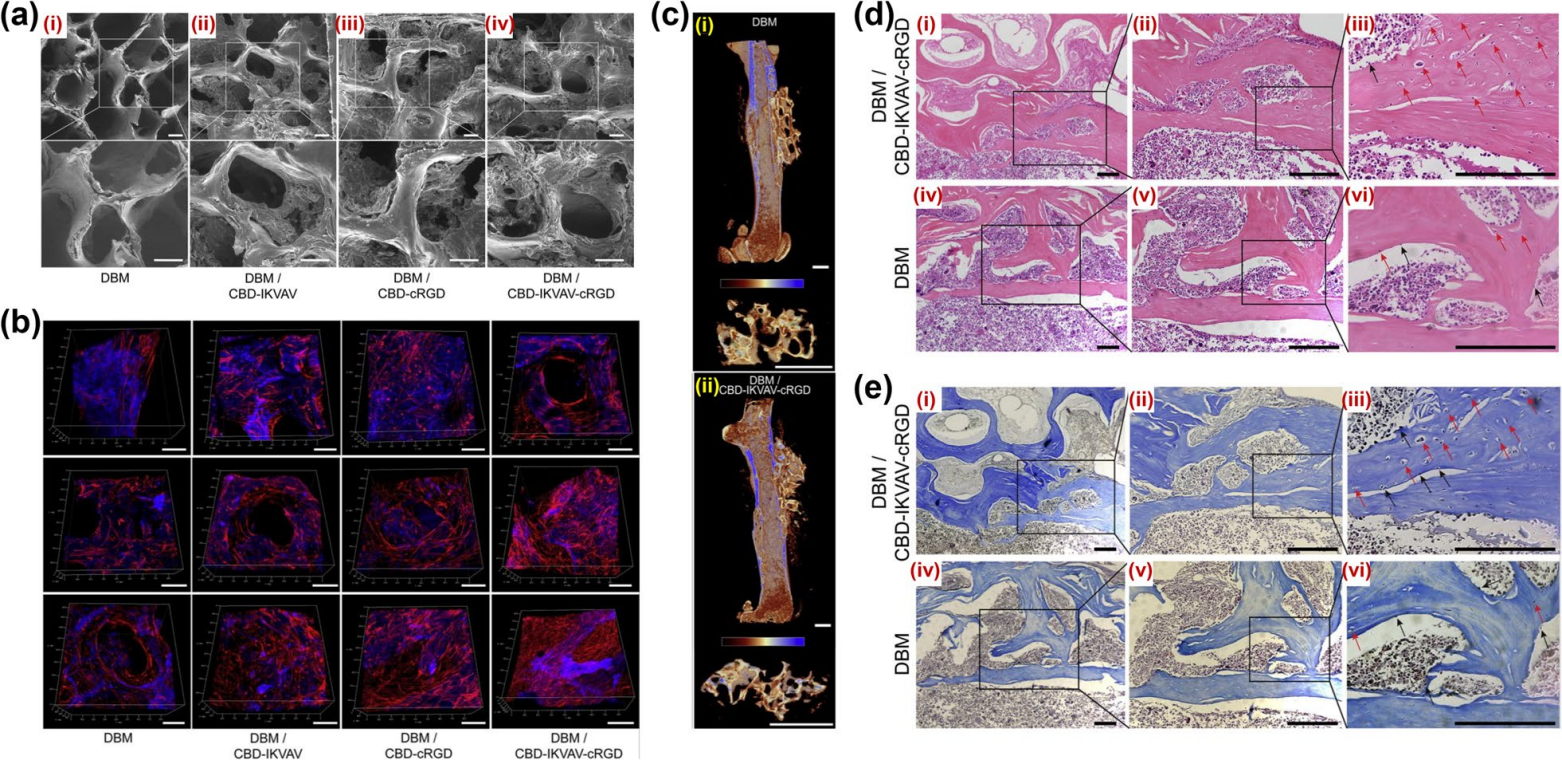
The composite scaffold based on DBM has the advantage of osteogenic differentiation. **a** Electron microscope scanning image of the morphological characteristics of various composite materials; **b** In vitro culture experiments of MSCs demonstrated that compared with pure DBM scaffold, composite DBM scaffolds were more suitable for the colonization and proliferation of MSCs; **c**–**e** In vivo transplantation experiments confirmed that the composite scaffold has more advantages in vivo osteogenesis than the pure DBM scaffold. [107] Reproduced from Acta biomaterialia, 85, Luo K, Gao X, Gao Y, Li Y, Deng M, Tan J, et al., Multiple integrin ligands provide a highly adhesive and osteoinductive surface that improves selective cell retention technology, 106–16, 2019, with permission from Elsevier

There are relatively few researches on the impact of scaffold mechanics on MSCs differentiation, and most of the studies focused on the mechanical properties, morphology, biocompatibility, bioactivity and biodegradability of scaffold materials as well as their supporting effects on the growth and proliferation of MSCs. MSCs combined with biological scaffold implantation can further promote the homing of MSCs cells and increase the survival rate of transplanted cells, which has attracted wide attention [92, 93]. The selection of scaffold materials has a crucial impact on the function of scaffolds [94, 95]. Bone defects caused by bone dystrophy increase the risk of fracture, therefore, more emphasis is placed on the mechanical properties of biological scaffolds in the selection of biological scaffolds [96]. For osteoporotic bone defects, MSCs combined with 3D scaffolds not only provide certain mechanical strength, but also provide a favorable regeneration microenvironment for related cells and factors, which is conducive to the healing of bone defects [97, 98]. The chemical composition and crystal structure of β-tricalcium phosphate are similar to those of natural bone minerals, and has great mechanical strength, bone conduction and bone induction effect. The scaffolders constructed by tricalcium β-phosphate have superior biocompatibility, and have ability to promote the osteogenic differentiation of MSCs [99–102]. It was found that the bone defects of MSCs combined with tricalcium β-phosphate scaffolds could be repaired better, and the osteogenesis ability was significantly improved, which was detected by X-ray and micro-CT in animal model of osteoporotic bone defect [103]. Besides, decalcified bone matrix (DBM) is a classic natural material for repairing bone defects, with superior porosity and adhesion rate, and can be gradually degraded and absorbed with the progress of osteogenesis, without producing acid metabolites harmful to osteogenesis [104, 105]. Wang et al. implanted MSCs combined with DBM after osteogenesis induction in vitro into the bone defect of ovariectomized osteoporosis rabbits. After transplantation, X-ray and Micro-CT indicated that MSCs combined with DBM scaffold formed dense tissue, which had a superior repair effect on bone defect, and showed that the osteogenic activity was enhanced, significantly promoting bone regeneration at the defect compared with single DBM [106]. The combination of DBM-based scaffolders and multiple factors can enhance the performance of scaffolds (Fig. 4). In addition, bone regeneration units composed of BMSCs-loaded VEGF/DBM-GelMA/HAMA exhibit significant osteogenic effects in vivo, which is a potential strategy for large bone repairmen [104].

**Fig. 5:**
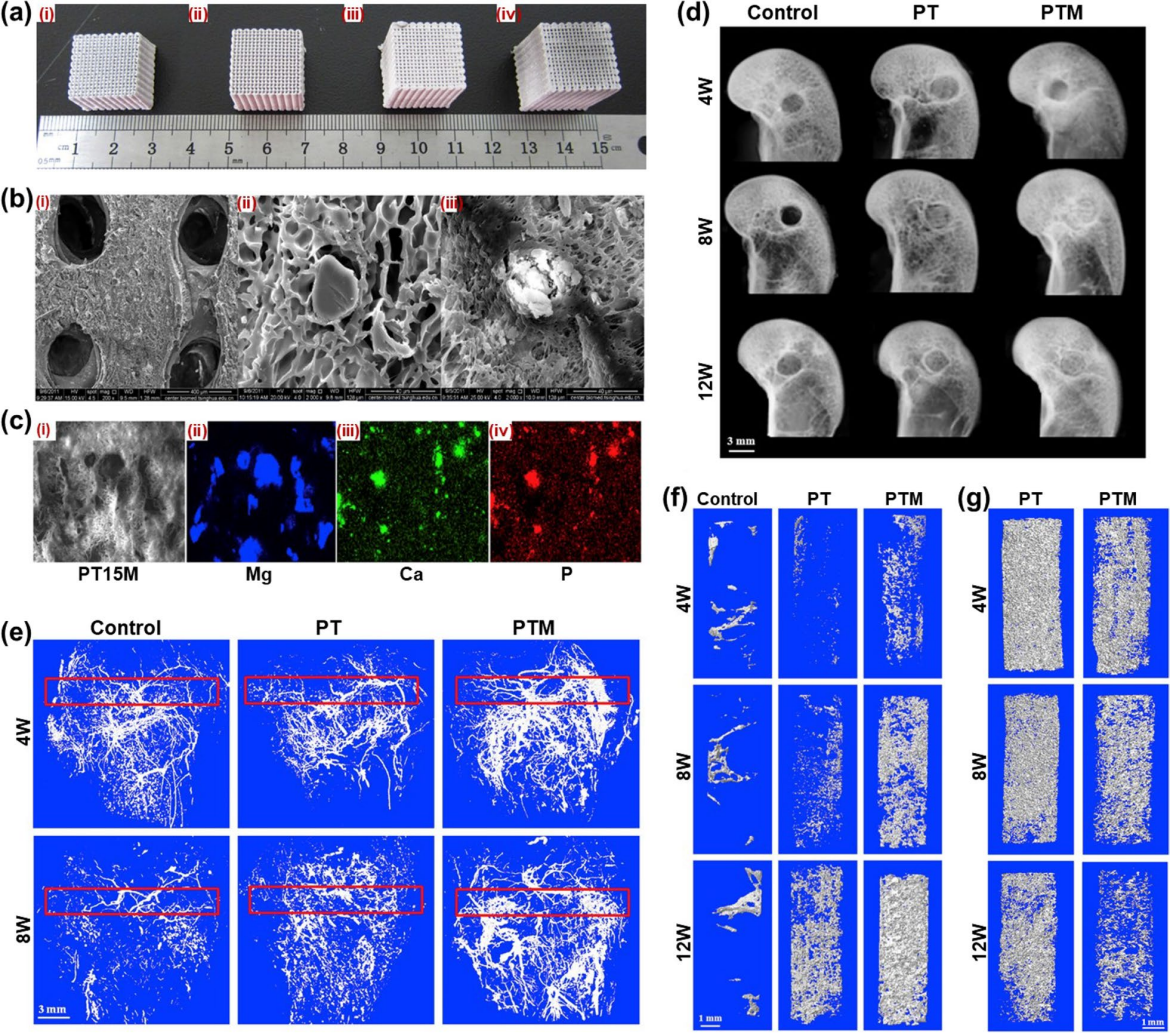
PLGA/tricalcium phosphate scaffold combined with Mg is more conducive to neovascularization and promote new bone formation. **a** General appearance of PLGA/tricalcium phosphate (PT) scaffolds with different Mg contents (PTM); **b** The microscopic morphology of scaffold and the particles distribution (Mg, Ca, P); **d**–**f** Bone formation was significantly increased after the transplantation of PTM scaffold and was more conducive to angiogenesis in vivo; **g** Residual status of bone tunnel scaffold in PT transplantation and PTM transplantation. In vivo transplantation of PTM scaffold has a high degradation rate. [111] Reproduced from Biomaterials, 197, Lai Y, Li Y, Cao H, Long J, Wang X, Li L, et al., Osteogenic magnesium incorporated into PLGA/TCP porous scaffold by 3D printing for repairing challenging bone defect, 207–19, 2019, with permission from Elsevier

Moreover, it has indicated that combine MSCs with poly lactic-co-glycolic acid (PLGA) scaffolds have also presented superior osteogenic effects in rat models with osteoporotic bone defects [108]. Different properties and types of PLGA can be obtained by adjusting the proportion of monomers in copolymers or by combining with special factors. PLGA combines the properties of lactic acid and glycolic acid and has a completely decomposed ester group with controllable degradation rate [109, 110]. As shown in Fig. 5, PTM scaffolds were synthesized by combining PLAG/tricalcium phosphate scaffolds with Magnesium. PTM scaffolds not only provided structural basis for neovascularization and promoted the formation of new bone, but also had high degradation rate in vivo, reducing the possibility of adverse reactions of scaffolds [111]. The repair process of bone defects is complex and difficult, so the selection of bone repair materials in bone tissue engineering as an alternative to traditional bone reconstruction methods is important. Therefore, further in vivo research is needed to investigate the integration ability of scaffolds, angiogenesis in bones, possible inflammatory reactions, and long-term stability in vivo based on the repair of bone defects, and to better translate laboratory data into clinical related strategies, providing new ideas for bone defect repair.

**Fig. 6:**
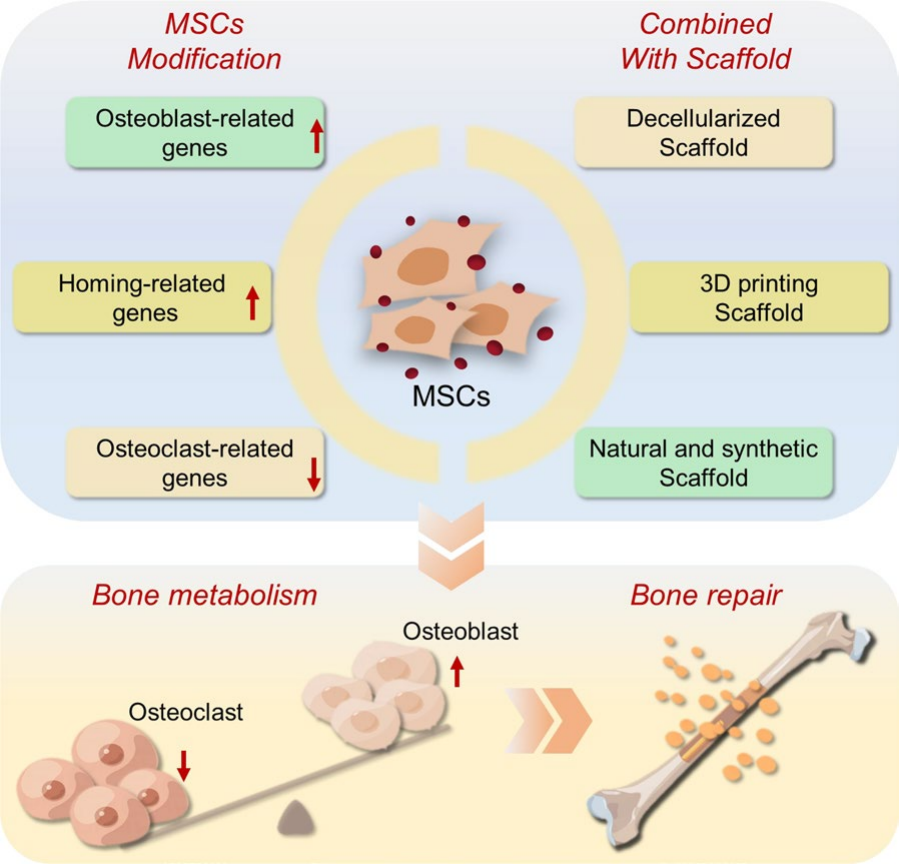
Therapeutic strategy of MSCs transplantation. By Figdraw (www.figdraw.com)

With the development of regenerative medicine and the deepening of cell research, the transplantation of MSCs as a novel treatment strategy for osteoporosis has been widely valued (Fig. 6). Considering the limitation of low survival rate and homing rate of MSCs, MSCs combined with scaffolds were applied in osteoporotic bone defects to enhance the homing effect of MSCs, induce MSCs to differentiate into osteoblasts and provide mechanical support, so that the combination of MSCs and scaffolds has occupied a place in the treatment and research of osteoporotic bone defects. However, on the basis of ensuring sufficient mechanical strength and biocompatibility, how the scaffold can further improve the adhesion of MSCs, promote growth and assist the directional differentiation of MSCs still needs further exploration.

## Conclusions

Hepatic osteodystrophy not only seriously disturbs the living quality of patients, increases the risk of fracture, but also brings a heavy burden to society. The current drug therapy cannot fundamentally reverse the bone loss of patients with HOD, and will bring a series of side effects to patients. As a kind of cells with differentiation potential, MSCs exert an extremely significant role in bone homeostasis. At present, numerous experiments have confirmed that MSCs have ability to mediated bone balance, accelerate bone formation, and have infinite potential in the treatment of osteoporosis. In addition, the therapeutic effect of the MSCs-loaded scaffold materials in the treatment of osteoporotic bone defects has also been affirmed, but more superior biological scaffolds still need to be further explored. Although the application of MSCs transplantation in HOD disease models has been rarely reported, the therapeutic effect of MSCs transplantation in osteoporosis has been confirmed, so MSCs transplantation therapy can be regarded as a promising preclinical and clinical direction for the treatment of hepatic osteodystrophy or bone defect caused by HOD.

## Data Availability

The datasets generated during and/or analyzed during the current study are available from the corresponding author on reasonable request.

## Abbreviations

HOD: Hepatic osteodystrophy
CLD: Chronic liver disease
MSCs: Mesenchymal stromal cells
BMD: Bone mineral density
IGF-1: Insulin-like growth factor-1
DBM: Decalcified bone matrix
PLGA: Poly lactic-co-glycolic acid
